# The human immunodeficiency virus type 1 Vpr protein and its carboxy-terminally truncated form induce apoptosis in tumor cells

**DOI:** 10.1186/1475-2867-9-20

**Published:** 2009-08-12

**Authors:** Mizuho Nonaka, Yoshie Hashimoto, Shin-nosuke Takeshima, Yoko Aida

**Affiliations:** 1 Viral Infectious Diseases Unit, RIKEN, 2-1 Hirosawa, Wako, Saitama 351-0198, Japan; 2 Japanese Foundation of AIDS Prevention, Tokyo, Japan

## Abstract

The human immunodeficiency virus type 1 (HIV-1) accessory protein Vpr induces apoptosis after cell cycle arrest at the G_2 _phase in primate cells. We have reported previously that C81, a carboxy-terminally truncated form of Vpr, interferes with cell proliferation and results in apoptosis without G_2 _arrest. Here, we investigated whether this property of Vpr and C81 could be exploited for use as a potential anticancer agent. First, we demonstrated that C81 induced G_1 _arrest and apoptosis in all tumor cells tested. In contrast, Vpr resulted in G_2 _arrest and apoptosis in HeLa and 293 T cells. Vpr also suppressed the damaged-DNA-specific binding protein 1 (DDB1) in HepG2 cells, thereby inducing apoptosis without G_2 _arrest. G_2 _arrest was restored when DDB1 was overexpressed in cells that also expressed Vpr. Surprisingly, C81 induced G_2 _arrest when DDB1 was overexpressed in HepG2 cells, but not in HeLa or 293 T cells. Thus, the induction of Vpr- and C81-mediated cell cycle arrest appears to depend on the cell type, whereas apoptosis was observed in all tumor cells tested. Overall, Vpr and C81 have potential as novel therapeutic agents for treatment of cancer.

## Background

The human immunodeficiency virus type 1 (HIV-1) accessory gene *vpr *encodes a 15-kDa protein of 96 amino acids. Vpr has multiple biological functions, including nuclear import of the preintegration complex [1-4], transactivation of the viral promoter [5], regulation of splicing [6-8], induction of apoptosis, and cell cycle arrest at the G_2 _phase [9,10]. The induction of cell cycle arrest at G_2 _is particularly important because the transcriptional activity of the HIV-1 long terminal repeat is most active in G_2 _[11,12], which leads to efficient virus production. Moreover, the cytostatic activity of Vpr is well conserved among the primate lentiviruses [13,14]. Thus, Vpr-mediated cell cycle arrest may play an important role in the life cycle of HIV-1.

Vpr induces G_2 _arrest by activating the ataxia telangiectasia-mutated and Rad3-related protein (ATR) [15,16]. ATR is a sensor of replication stress that is activated by the stalling of replication forks after various cellular insults, such as deoxyribonucleotide depletion, topoisomerase inhibition, or UV light induced DNA damage [17]. A recent report suggests that Vpr induces ATR activation, leading to the induction of G_2 _arrest [18]. Additionally, the Vpr-binding protein (VprBP), which was isolated as a Vpr coprecipitating protein, acts as a substrate specificity determinant in a Cul4- and damaged-DNA-specific binding protein 1 (DDB1)-based E3 ubiquitin ligase complex [19-22]. Thus, VprBP was renamed the DDB1- and Cul4A-associated factor 1 (DCAF1). Several groups have recently confirmed that Vpr physically associates with DCAF1 and that Vpr is capable of binding to a larger complex consisting of Cul4A, DDB1, and DCAF1 [18,23-27]. The interaction between Vpr and DDB1 could cause the accumulation of damaged DNA by preventing DNA repair. The failure of DDB1 to bind DNA and mediate the repair of DNA lesions generated during S phase may be sensed by the cell as DNA damage, which would activate ATR, resulting in G_2 _arrest followed by apoptosis [26]. However, Vpr mutants such as Vpr(1-78) or Vpr(R80A) are able to bind to DCAF1, but are unable to induce G_2 _arrest [24,28]. The association of Vpr with the E3 ligase complex is necessary but not sufficient to induce G_2 _arrest, suggesting that Vpr may induce the ubiquitination and degradation of an unknown cellular factor.

Several researchers have demonstrated that Vpr is capable of inducing cell cycle arrest and apoptosis in cancer cell lines, including those defective for some tumor suppressor genes and DNA repair genes [29,30]. In addition, Siddiqui and colleagues [31] have reported that Vpr is involved in DNA damage that leads to the disruption of the cell cycle. These results suggest that Vpr mimics the action of the anticancer agent cisplatin. Furthermore, it has also been reported that Vpr has anti-melanoma activity *in vivo *[32,33]. These studies suggest that Vpr has the potential for therapeutic application as a tumor suppressor. Interestingly, we have found that a carboxy-terminally truncated form of the Vpr, C81, induces G_1 _arrest, but not G_2 _arrest or apoptosis, via disruption of mitochondrial function [34,35]. We have also shown that caspase-3 activity induced by C81 is higher than that induced by Vpr in human cells [35]. Thus, our results suggest that C81 may be a strong inducer of tumor cell apoptosis and may have potential as an anti-tumor agent.

In this study, we examined whether C81, as well as Vpr, induced cell cycle arrest and apoptosis in several tumor cell lines, and examined the expression patterns of factors associated with apoptosis and cell cycle arrest.

## Results

### The expression of Vpr and C81 induces apoptosis in tumor cells

**Figure 1:**
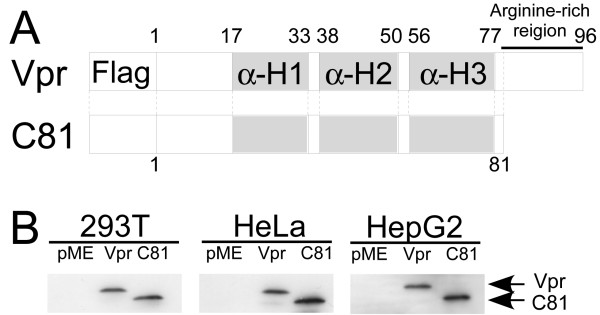
**Construction and expression of Vpr and C81**. (A) Plasmids containing cDNA for Vpr and C81 were generated from HIV-1_NL4-3_. Schematic presentation of the predicted first α-helical domain (αH1), second α-helical domain (αH2), third α-helical domain (αH3) and the arginine rich region of Vpr are indicated. (B) Western blotting of wild-type Vpr and C81. HeLa, HepG2, and 293 T cells were transfected with pME18Neo encoding Flag-tagged wild-type Vpr or C81, or the empty vector control pME18Neo-Flag, together with pSV-β-galactosidase. Transfected cells were collected and lysed 48 h after transfection. Lysates with equal β-galactosidase activity were subjected to Western blot analysis using anti-Flag.

Vpr has previously been demonstrated to induce G_2 _cell cycle arrest as well as apoptosis in a number of tumor cells [29,30]. Previously, we found that C81, a carboxy-terminally truncated form of Vpr, is a strong inducer of apoptosis with G_1 _arrest, but not G_2 _arrest, of the cell cycle, and that the apoptotic activity of C81 is stronger than that of Vpr in human cells [35]. Based on the growth arresting and apoptosis inducing properties in tumor cells, C81 and Vpr have potential therapeutic applications for treating cancer. To further investigate this potential, we examined whether Vpr and C81 can induce apoptosis in tumor cells. We transiently transfected two human tumor cell lines, HeLa and HepG2, as well as 293 T cells, with a pME18Neo expression vector encoding Flag-tagged wild-type Vpr, C81, or the empty vector pME18Neo-Flag as a control. We examined the expression of Vpr and C81, 48 h post-transfection by Western blot analysis using the anti-Flag M2 monoclonal antibody. Fig. 1 shows protein bands with molecular masses that are consistent with the calculated masses of the sequences of Flag-tagged Vpr and Flag-tagged C81.

**Figure 2:**
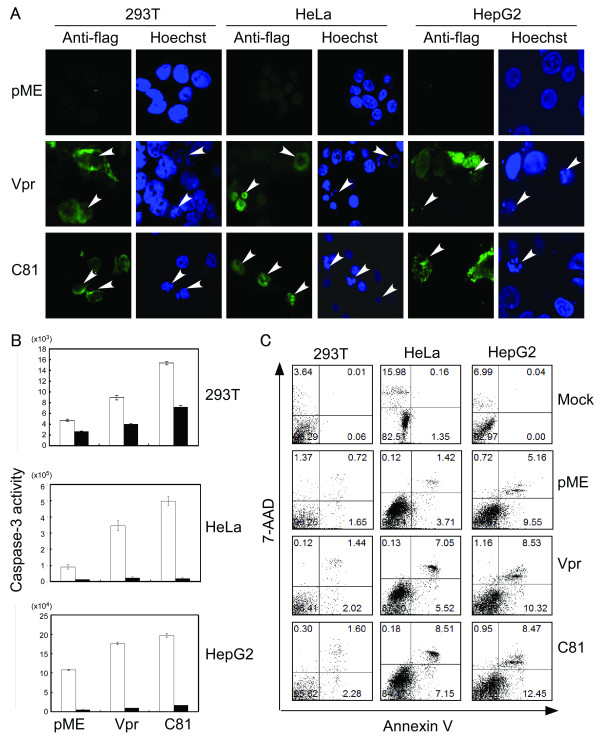
**Vpr and C81 induce apoptosis in tumor cells**. (A) HeLa, HepG2, and 293 T cells were transfected with pME18Neo encoding Flag-tagged wild-type Vpr or C81, or the control pME18Neo-Flag. At 48 h post-transfection, cells were fixed and stained with anti-Flag, followed by Alexa-488-conjugated anti-mouse IgG. Finally, cells were stained with Hoechst 33258 to monitor the nuclear morphology. Apoptotic bodies (arrowheads) were identified using confocal laser scanning microscopy. (B) HeLa, HepG2, and 293 T cells were transfected with the pME18Neo plasmid encoding Flag-tagged wild-type Vpr or C81, or the control pME18Neo-Flag, together with pSV-β-galactosidase. Cells were treated with (filled columns) or without (open columns) inhibitors of caspase-3. At 30 h (293 T), 36 h (HeLa), or 48 h (HepG2) post-transfection, caspase-3 activity was measured and normalized to the β-galactosidase activity. Each column and error bar represents the mean ± SD of measurements from three samples. (C) HeLa, HepG2, and 293 T cells were transfected with pME18Neo encoding Flag-tagged wild-type Vpr or C81, or the control pME18Neo-Flag with or without the GFP expression vector pEGFP-N1. GFP was used as a reporter to discriminate between transfected and untransfected cells. At 47 h (293 T), 38 h (HeLa), or 58 h (HepG2) post-transfection, cells were stained with PE-Annexin V and 7-AAD to identify apoptotic cells, or anti-mouse IgG_2b_-PE (Nippon BD Company Ltd) and 7-AAD as a negative control. The percentages of Annexin V-positive and 7-AAD negative cells relative to GFP-positive cells indicate the level of Vpr or C81 associated apoptosis.

Next, we detected apoptotic cells by monitoring fluorescence after staining the cells with Hoechst 33258. HeLa, HepG2, and 293 T cells were transfected with the pME18Neo empty vector, or the expression plasmids for Vpr or C81. At 48 h post-transfection, cells were fixed and stained with anti-Flag followed by Alexa-488-conjugated anti-mouse IgG. To monitor nuclear morphology, cells were stained with Hoechst 33258. The cells that expressed either Vpr or C81 had condensed chromatin, a hallmark of cells undergoing apoptosis (Fig. 2A). Furthermore, we assessed apoptosis in Vpr- and C81-expressing cells by measuring the activity of caspase-3, which plays an essential role in the induction of apoptosis (Fig. 2B). Caspase-3 activity was significantly higher in the Vpr- and C81-expressing cells compared to the control vector transfected cells. Caspase-3 activity was approximately two-five fold higher than in the cells transfected with the control pME18Neo empty vector. Interestingly, caspase-3 activity was significantly higher in cells that had been transfected with the C81 expression vector, compared to cells transfected with the Vpr expression vector. Conversely, treatment with the caspase-3 specific inhibitor, Z-DEVD-FMK, suppressed the activity of caspase-3 in all transfected cells, including cells transfected with the control pME18Neo empty vector. These results strongly suggested that Vpr and C81 can induce apoptosis in the HepG2 and HeLa cell lines.

The apoptotic activity of Vpr and C81 was further quantified with flow cytometry by combined staining of transfected samples with phycoerythrin-(PE)-Annexin V and 7-amino-actinomycin D (7-AAD) (Fig. 2C). A prominent event in early apoptosis is the exposure of phosphatidylserine (PS) on the outer leaflet of the cell membrane. Cell surface exposed PS can be specifically detected with PE-Annexin V. At late stages of apoptosis, or in necrosis, cell membrane integrity is lost, allowing entry of the DNA-binding dye 7-AAD into cells. The population of Annexin V positive and 7-AAD negative apoptotic cells among cells expressing Vpr or C81 reached to approximately 5.52% and 7.15% in HeLa cells, respectively, and 10.32% and 12.45% in HepG2 cells, respectively. These values were much higher than in cells that had been transfected with the control pME18Neo empty vector or mock transfected (3.71% and 1.35% in HeLa cells, respectively, and 9.55% and 0% in HepG2 cells, respectively). Interestingly, the populations of apoptotic cells in both tumor cell lines were significantly higher in cells transfected with the C81 expression vector, compared to cells transfected with the Vpr expression vector. Similar results were obtained with 293 T cells. Only a small population of 293 T cells transfected with either Vpr, C81, or the control vector bound Annexin V (2.02%, 2.28%, and 1.65%, respectively). Since these results show the same trends as the Hoechst 33258 staining (Fig. 2A) and the caspase-3 activity (Fig. 2B), we conclude that Vpr and C81 induce apoptosis in the human tumor cell lines HepG2 and HeLa.

### Vpr and C81 induce apoptosis and caspase-9 activity

**Figure 3:**
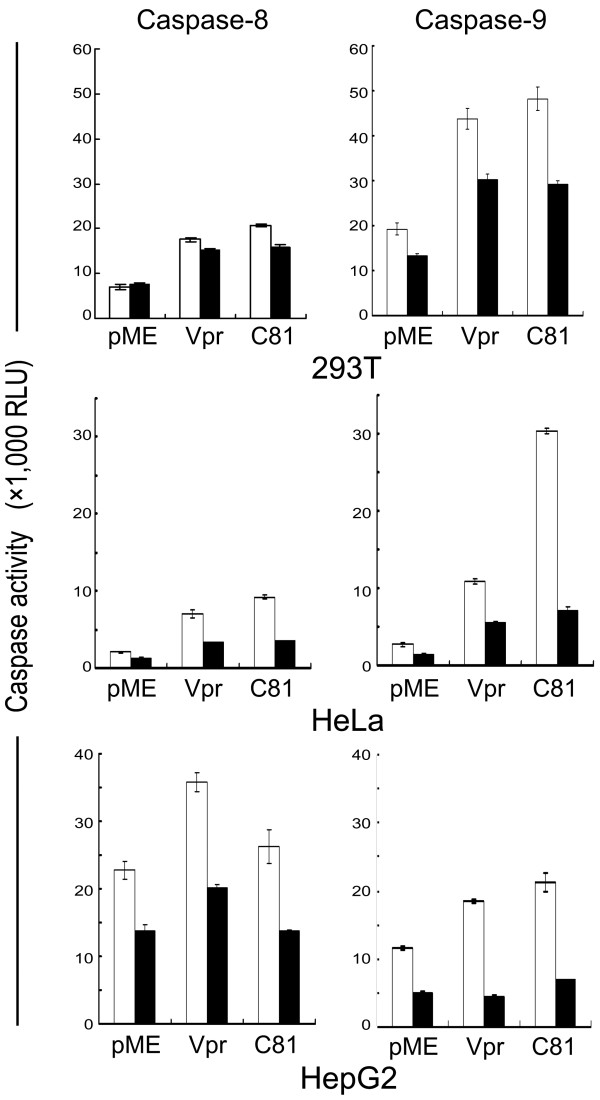
**Vpr and C81 induce apoptosis increased caspase-9 activity**. HeLa, HepG2, and 293 T cells were transfected with pME18Neo encoding Flag-tagged wild-type Vpr or C81, or the control pME18Neo-Flag, together with pSV-β-galactosidase before treatment with (filled columns) or without (open columns) a caspase-8 or caspase-9 inhibitor. At 30 h (293 T), 36 h (HeLa), or 48 h (HepG2), post-transfection, caspase-8 or caspase-9 activity was measured and normalized to the β-galactosidase activity. Columns and error bars represent the mean ± SD of measurements from three samples.

Vpr and C81 are reported to target mitochondria to induce apoptosis [10,34]. To examine whether Vpr and C81 target mitochondria in tumor cells, we measured caspase-8 and caspase-9 activity. Caspase-8 is activated by FasL and tumor necrosis factor-α (TNF-α), whereas caspase-9 is activated by the disruption of mitochondrial function. In tumor cells, as well as 293 T cells, caspase-9 activity induced by Vpr or C81 was higher than caspase-8 activity (Fig. 3). Caspase-9 activity in Vpr or C81-transfected cells was significantly higher than in the control vector transfected cells (p < 0.01). In addition, although apoptosis induced by Vpr or C81 was significantly inhibited when inhibitors of caspase-9 were added to the Vpr- or C81-transfected cells (p < 0.01), a caspase-8 inhibitor only slightly inhibited apoptosis in these transfected cells. These results suggest that both Vpr and C81 induce apoptosis via the activation of caspase-9.

### Induction of cell cycle arrest by Vpr and C81 depends on the cell type

**Figure 4:**
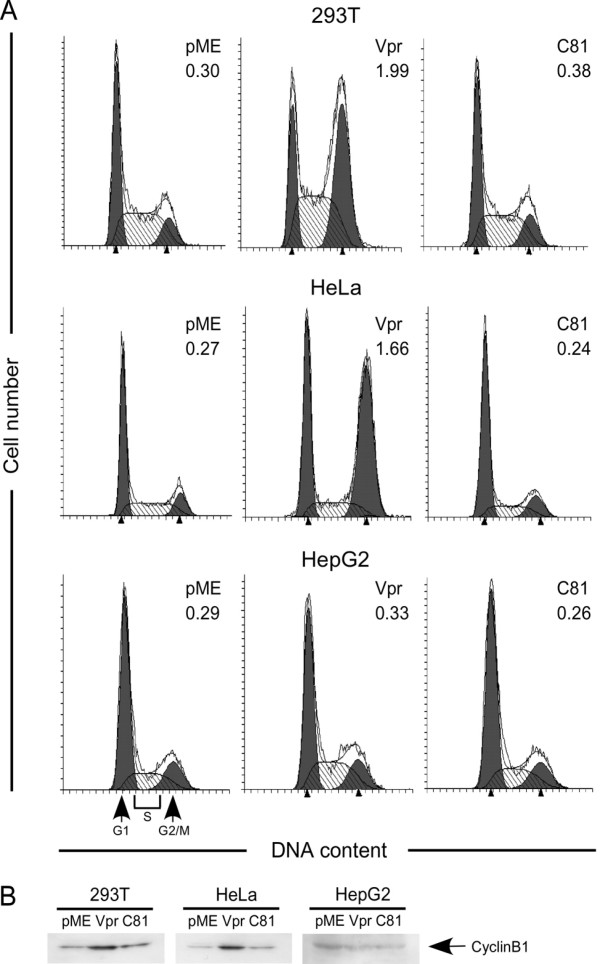
**Induction of cell cycle arrest by Vpr or C81 is dependent on the cell type**. (A) HeLa, HepG2, and 293 T cells were transfected with pME18Neo encoding Flag-tagged wild-type Vpr or C81, or the control pME18Neo-Flag, together with the GFP expression plasmid pEGFP-N1. At 48 h post-transfection, cells were fixed and stained with PI. GFP-positive cells were analyzed by flow cytometry using CELL Quest for acquisition and ModFit LT for quantitative analysis of DNA content. Arrows indicate the peaks of cells at the G_1 _and G_2_/M phases. The G_2_/M:G_1 _ratios are indicated at the upper right in each graph. (B) HeLa, HepG2, and 293 T cells were transfected with pME18Neo encoding Flag-tagged wild-type Vpr or C81, or the control plasmid pME18Neo-Flag, together with pSV-β-galactosidase. At 48 h post-transfection, the cells were collected and lysed. Lysates with equal β-galactosidase activity were subjected to Western blot analysis using a cyclin B1 specific antibody.

Vpr induces G_2 _cell cycle arrest in various human cells [10], whereas C81 induces G_1 _cell cycle arrest in human and rodent cells [34,35]. To determine whether Vpr and C81 induce cell cycle arrest in HeLa, HepG2, or 293 T cells, we transfected these cells with pME18Neo encoding Flag-tagged wild-type Vpr or C81, or the control plasmid pME18Neo-Flag, together with a small amount of a green fluorescent protein (GFP) expression vector, pEGFP-N1, to identify transfected cells. Cells were fixed and stained with propidium iodide (PI) 48 h post-transfection and analyzed by flow cytometry. We measured the DNA content of GFP-positive cells to determine the distribution of cells across the cell cycle (Fig. 4A). C81 failed to arrest the cell cycle at the G_2 _phase in HeLa, 293 T, or HepG2 cells. To confirm this result, we prepared total cell extracts and examined cyclin B1, which controls the cell cycle in the G_2 _phase, by Western blot analysis (Fig. 4B). C81 failed to induce accumulation of cyclin B1 in HeLa, 293 T, or HepG2 cells, suggesting that C81 failed to arrest the cell cycle at the G_2 _phase in these cells. In contrast, transfection with Vpr led to an increase in the number of HeLa and 293 T cells in the G_2 _phase of the cell cycle, compared with cells that had been transfected with the control pME18Neo-Flag. Moreover, Vpr induced accumulation of cyclin B1 in HeLa and 293 T cells. However, Vpr was unable to arrest the cell cycle at the G_2 _phase, or to induce accumulation of cyclin B1, in HepG2 cells (Fig. 4).

### DDB1 overexpression enables G2 cell cycle arrest by Vpr and C81

**Figure 5:**
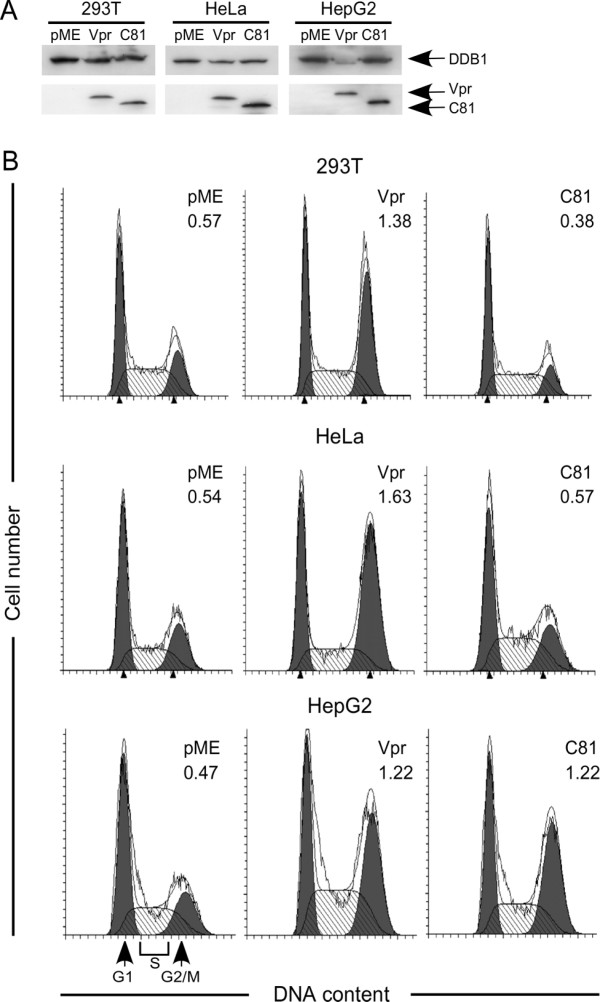
**Overexpression of DDB1 induces G_2 _cell cycle arrest by Vpr and C81**. (A) HeLa, HepG2, and 293 T cells were transfected with pME18Neo encoding Flag-tagged wild-type Vpr or C81, or the control pME18Neo-Flag, together with pSV-β-galactosidase. At 48 h post-transfection, the cells were collected and lysed. Lysates with equal β-galactosidase activity were subjected to Western blotting with either a DDB1 specific antibody or the Flag-specific MAb M2. (B) HeLa, HepG2, and 293 T cells were transfected with pME18Neo encoding Flag-tagged wild-type Vpr or C81, or the control pME18Neo-Flag, together with the pDDB1-IRES-GFP expression plasmid and pSV-β-galactosidase. Lower panel: 48 h post-transfection, cells were fixed and stained with PI. GFP-positive cells were analyzed by flow cytometry using CELL Quest for acquisition and ModFit LT for quantitative analysis of DNA content. Arrows indicate the peaks of cells at the G_1 _and G_2_/M phases. The G_2_/M:G_1 _ratio is indicated at the upper right in each graph.

It has recently been reported that Vpr targets the DCAF1 adaptor of the Cul4A-DDB1 ligase to induce cell cycle arrest at the G_2 _phase [28]. Thus, we examined the expression levels of DDB1 in various cell lines, including HepG2 cells, which do not arrest at G_2 _when transfected with Vpr (Fig. 5A). Interestingly, Western blot analysis revealed that Vpr, but not C81, could inhibit DDB1 expression in HepG2 cells. In contrast to Vpr, C81 had no effect on the expression of DDB1 in HepG2 cells.

To determine the effect of DDB1 in HepG2 cells, we constructed a DDB1 expression vector, pDDB1-IRES-GFP, which was co-transfected with either the Vpr or C81 expression vector. As shown in Fig. 5B, overexpression of DDB1 resulted in cell cycle arrest at G_2 _in HepG2 cells transfected with Vpr. Overexpression of DDB1 had no effect on the induction of G_2 _arrest by Vpr in HeLa or 293 T cells. This result clearly suggests that DDB1 plays an essential role in Vpr-induced cell cycle arrest. Strikingly, C81, which was completely inactive for G_2 _arrest in all cell lines tested, was able to induce G_2 _arrest in HepG2 cells overexpressing DDB1, but not in HeLa or 293 T cells overexpressing DDB1. These results suggest that C81 can cause G_2 _arrest independent of its ability to induce apoptosis via G_1 _arrest of the cell cycle, although the former activity is cell type dependent.

## Discussion

The results of the present study show that C81 and Vpr induce apoptosis via the activation of caspase-9 in tumor cells such as HeLa, HepG2, and 293 T cells. A previous report indicated that Vpr and C81 induced apoptosis in rodent cell lines [34], suggesting that the induction of apoptosis by Vpr and C81 may be ubiquitous. Vpr displayed cell type specificity in terms of induction of G_2 _arrest but not apoptosis. Vpr resulted in apoptosis that was coupled with G_2 _arrest in HeLa and 293 T cells, whereas Vpr induced apoptosis without G_2 _arrest in HepG2 cells. Similarly, we previously reported that, in rodent cell lines, Vpr fails to induce cell cycle arrest at G_2 _[34]. The present study also shows that DDB1 is required for Vpr-mediated G_2 _arrest. Notably, the expression of Vpr, but not C81, suppressed DDB1 expression in HepG2 cells where it induced apoptosis without G_2 _arrest. In contrast, Vpr induced G_2 _arrest and apoptosis in HeLa and 293 T cells.

Interestingly, G_2 _arrest was restored when DDB1 was overexpressed by transfection during Vpr expression. Additionally, C81, which was unable to induce G_2 _arrest in all cells tested, induced G_2 _arrest when overexpressed with DDB1 in HepG2 cells, but not in HeLa or 293 T cells. These results indicate that the induction of cell cycle arrest at the G_2 _phase by Vpr or C81 may depend on the level of DDB1 expression in Vpr- and C81-transfected cells. Finally, we demonstrated that the apoptotic properties of C81, as well as those of Vpr, are strong inducers of intra-tumoral apoptosis. These proteins hold great promise as the basis for an anti-cancer treatment, particularly since C81 caused apoptosis in the tumor cell line HepG2, in which Vpr failed to induce G_2 _arrest.

Vpr binding to the adenine nucleotide transporter (ANT) protein of the inner mitochondrial membrane was previously shown to induce apoptosis through permeabilization of the mitochondrial outer membrane [9]. Vpr has also been reported to activate caspase-9 and apoptosis in other human tumor cell lines [29]. Moreover, C81 induced cell cycle arrest at the G_1 _phase and apoptosis through activation of caspase-3 and caspase-9 in rodent cells, which was associated with the release of cytochrome *c *from mitochondria into the cytosol and disruption of the mitochondrial transmembrane potential [34]. We have demonstrated here that caspase-9 is activated during Vpr and C81 induced apoptosis, confirming that the induction of apoptosis by Vpr and C81 is caused by mitochondrial dysfunction.

Analysis of the three-dimensional structure of Vpr by nuclear magnetic resonance (NMR) revealed three well-defined α-helixes, amino acids 17-33 (αH3), 38-50 (αH2), and 56-77 (αH3), which are surrounded by flexible amino- and carboxy-terminal domains [36]. Siddiqui and colleagues have suggested that the αH2 domain in Vpr is responsible for DNA damage [31]. Interestingly, Vpr Delta (37-50) failed to induce cell cycle arrest or apoptosis, or to induce Ku70 or Ku80 expression or suppress tumor growth. However, the peptide activated the HIV-1 LTR, resulting in its localization to the nucleus where it promoted nonhomologous end-joining [31]. C81 shares the αH2 domain with Vpr; thus, C81 may have the ability to induce DNA damage. In this study, both wild-type Vpr and C81 induced apoptosis that may be triggered by DNA damage involving DDB1.

Vpr stabilization was recently reported to be dependent on DDB1, suggesting that the Cul4A-DDB1-DCAF1 ubiquitin ligase is required to protect Vpr from proteasomal degradation [37]. Here, we demonstrated that, in HepG2 cells, Vpr was expressed stably despite a decrease in DDB1 expression. Therefore, further studies will be required to clearly define whether DDB1 protects Vpr from proteasomal degradation in HepG2 cells. Whether this downregulation of DDB1 occurs at the transcriptional or protein level, and whether the interaction of Vpr with the E3 ligase complex is involved in the biological activity should also be addressed in future studies.

The activation of G_2 _arrest by Vpr is induced via binding and, possibly, activation of the Cul4A-DDB1-DCAF1 ligase [28]. In addition, DCAF1 engages in simultaneous interactions with DDB1 and Vpr, leading to the formation of a ternary complex. The domain of Vpr that binds to DCAF1 was mapped to the leucine-rich ^60^LIRILQQLL^68 ^motif in the αH3 domain of Vpr [38]. The truncation of the C-terminal region of Vpr up to residue H78 or the replacement of arginine at position 80 by alanine did not affect the Vpr interaction with DCAF1 but affected the ability of Vpr to induce G_2 _arrest [18,24]. Similarly, our previous results [3,35,39,40] showed that C81, a carboxy-terminal truncated form of Vpr, was completely inactive for G_2 _arrest in all cells tested. Together, these experiments indicate that interactions between Vpr and DCAF1 are necessary but not sufficient for the induction of G_2 _arrest and that the carboxy-terminal domain of Vpr is likely to be required for the recruitment of a cellular protein whose ubiquitination and degradation leads to G_2 _arrest. Interestingly, we found that C81 resulted in G_2 _arrest when overexpressed with DDB1 in HepG2 cells, but not in HeLa or 293 T cells. This finding suggests that C81 may interact with DCAF1, causing G_2 _arrest via activation of the CUl4A-DDB1-DCAF1 ligase, when DDB1 is overexpressed in HepG2 cells. Additionally, the ability of C81 to induce apoptosis with G_1 _arrest may be a dominant function compared to induction of G_2 _arrest in almost all other cells. Moreover, the expression of Vpr suppressed DDB1 activity in HepG2 cells but not in HeLa cells or 293 T cells, resulting in the induction of apoptosis without G_2 _arrest. The overexpression of DDB1 succeeded in inducing Vpr-mediated G_2 _arrest in HepG2 cells, suggesting that DDB1 is a key component of Vpr-mediated G_2 _arrest. Thus, the induction of G_2 _arrest by Vpr and C81 may be dependent on the expression level of DDB1.

## Conclusion

Vpr has been reported to induce apoptosis in transformed cells and human tumor cell lines [29,30]. Furthermore, Vpr successfully induces the regression of murine melanoma tumors *in vivo *[33]. Because our data indicate that Vpr and C81 induce apoptosis in tumor cell lines, these factors could be potentially useful in the development of novel therapeutic treatments for cancer.

## Materials and methods

### Plasmid construction

The expression vector pME18Neo encoding a Flag-tagged wild-type Vpr or C81, and the control vector pME18Neo have been described previously [40,41]. A red-shifted variant of wild-type GFP that has been modified for brighter fluorescence was encoded by pEGFP-N1 and used as a reporter to identify transfected cells [42]. Bacterial β-galactosidase encoded by pSV-β-galactosidase was included for normalization of the transfection efficiency [40]. The mRNA of human *DDB1 *was amplified by RT-PCR from RNA derived from Jurkat cells. RT was performed with an oligo-dT primer and PCR was performed using the following primers: 5'-gcggccgcacatgtcgtacaactacg-3' and 5'-gtcgacgctaatggatccgagttagc. The resulting amplicon served as the template for a second PCR reaction using the primers 5'-ataagaatgcggccgcacatgtcgt-3' and 5'-tggccgacgtcgacgctaatggatc-3'. The second PCR product was subcloned between the *Not*I-*Sal*I sites of pIRES-hrGFP-2a (Stratagene, La Jolla, CA), generating pDDB1-IRES-GFP.

### Cell lines and transfection

HeLa cells, which are human epithelial cervical carcinoma cells [43,44], and 293 T cells that stably express the large T-antigen of simian sarcoma virus 40 [45] were maintained in Dulbecco's modified Eagle's medium (DMEM) supplemented with 10% heat-inactivated fetal calf serum (FCS), penicillin (100 U/ml), and streptomycin (100 μg/ml). HepG2 cells, which were derived from a well differentiated hepatocellular carcinoma [46,47] were obtained from the RIKEN Bio Resource Center (Ibaraki, Japan) and maintained in DMEM supplemented with 10% heat-inactivated FCS, penicillin (100 U/ml), streptomycin (100 μg/ml), and 0.1 mM non-essential amino acids. Transfection of cells was performed using FuGENE HD (Roche Diagnostics, Basel, Switzerland) according to the manufacturer's instructions.

### Western blotting

At 24 or 48 h post-transfection with the indicated plasmids and pSV-β-galactosidase, cells were washed with phosphate-buffered saline (PBS) and divided into two aliquots. One aliquot was subjected to an assay for β-galactosidase activity with the FluoReporter LacZ/Galactosidase Quantitation kit (Molecular Probes, Eugene, OR), to monitor the transfection efficiency. The other aliquot of cells was lysed for 10 min on ice in 10 mM Tris-HCl (pH 8.0), 150 mM NaCl, 5 mM EDTA, 1% Triton X-100, 0.1% SDS, 0.1% desoxycholate supplemented with a protease inhibitor cocktail (Roche Diagnostics, Basel, Switzerland) and phosphatase inhibitor cocktail1 (Sigma, St. Louis, Mo). Lysates were centrifuged for 10 min at 20,000 g, mixed with sample buffer for SDS-PAGE and then boiled for 5 min. Protein concentrations were determined with a BCA protein assay kit (Pierce, Rockford, IL), with bovine serum albumin (BSA) as the standard. Aliquots with equal β-galactosidase activity were examined by Western blotting as described previously [35,39]. An anti-Flag MAb (M2) (Sigma, St. Louis, MO), anti-Cyclin-B1 (H-20) antibody (Santa Cruz biotechnology, Santa Cruz, CA), anti-DDB1 MAb (ZYMED Laboratories Invitrogen, Carlsbad, CA), horseradish-peroxidase (HRP)-conjugated sheep anti-mouse IgG (Amersham Bioscience, Uppsala, Sweden) and HRP-conjugated goat anti-rabbit IgGs (Cell Signaling Technology, Danvers, MA) were used for Western blotting. Signals were visualized after treatment of the membrane with SuperSignal West Pico chemiluminescent substrate (Pierce, Rockford, IL).

### Analysis of apoptosis

The activities of caspase-3, 8, and 9 were determined using caspase-3/CPP32, caspase-8/Flice or caspase-9/Mch6 fluorometric assay kits (Bio Vision, Mountain View, CA) according to the manufacturer's protocols. Briefly, 30 h or 40 h post-transfection, in the presence or absence of 2 μM caspase-3, 8, or 9 specific inhibitors (Bio Vision, Mountain View, CA), cells were harvested and lysed with cell lysis buffer. Each cell lysate was incubated with the substrate for 2 h at 37°C. Measurements were made using a multilabel cell counter (Model1420; Wallac Arvo; Parkin Elmer, Wellesley, MA).

Hoechst 33258 was used to visualize the condensation and clumping of chromatin. At 48 h post-transfection with pME18Neo encoding Flag-tagged wild-type Vpr, C81, or the control pME18Neo-Flag, HeLa cells that were grown on coverslips were fixed with 4% formaldehyde followed by 70% ethanol. Flag-tagged-Vpr or C81 was detected by indirect immunofluorescent staining with the Flag-specific MAb M2 (Sigma, St. Louis, MO), followed by Alexa-488-conjugated anti-mouse IgG. The cells were then stained with Hoechst 33258 (Sigma, St. Louis, MO). Apoptotic bodies were characterized using confocal laser scanning microscopy (FV 1000; Olympus, Tokyo, Japan).

To detect Annexin V positive apoptotic cells, we used flow cytometry. At the indicated times post-transfection, cell monolayers were washed with PBS, detached with trypsin and EDTA, and 1 × 10^5 ^cells were stained with 7-amino-actinomycin D (7-AAD) and Annexin V-PE (Nippon BD company Ltd, Japan) according to the manufacturer's instructions. Incubation of cell monolayers for 8 to 13 hours with actinomycin D (2 μg/ml) provided positive controls for apoptosis. For each sample, 10,000 GFP positive cells were analyzed using a FACS Calibur instrument (BD Biosciences, Japan) and the FCS Express version 3 software (De Novo software, CA).

### Analysis of the cell cycle

At 48 h post-transfection, cells were harvested and fixed with 1% formaldehyde and then 70% ethanol. Fixed cells were incubated in PBS that contained RNase A (50 μg/ml) at 37°C for 30 min and then stained with PI (50 μg/ml). The fluorescence of 20,000 cells was analyzed using a FACS Calibur instrument (Becton-Dickinson, Mountain View, CA) with the CELL Quest software (Becton-Dickinson, Mountain View, CA). Data were extracted after gating to eliminate cells in which GFP emitted strong fluorescence. Ratios of the numbers of cells in the G_1 _and G_2_/M phase (G_2_/M:G_1 _ratios) were calculated using the ModFit LT Software (Verity Software House, Topsham, ME).

### Statistical methodology

Statistical analyses were conducted using R version 2.8(1) [48].

## Competing interests

The authors declare that they have no competing interests.

## Authors' contributions

MN participated in the all experiments and drafted the manuscript. YH carried out the immunoassay, analysis of the cell cycle, measurements of caspase activity and helped to draft the manuscript. ST performed the statistical analysis, measured Annexin V-positive cells and helped to draft the manuscript. YA conceived of the study, and participated in its design, and coordination of experiments, and drafted the manuscript. All authors read and approved the final manuscript.
